# Cancer incidence and mortality in Barranquilla, Colombia. 2008-2012

**DOI:** 10.25100/cm.v49i1.3627

**Published:** 2018-03-30

**Authors:** Rusvelt Vargas Moranth, Edgar Navarro Lechuga

**Affiliations:** 1 Registro Poblacional de Cáncer de Barranquilla. Barranquilla, Colombia.; 2 Grupo de Investigación en Economía de la Salud, Universidad de Cartagena, Cartagena, Colombia.; 3 Grupo de Investigación Sanus Viventium, Barranquilla, Colombia; 4 Grupo de Investigación Proyecto UNI. Departamento de Salud Pública, Universidad del Norte, Barranquilla, Colombia.

**Keywords:** Cancer, incidence, mortality, population registries, Cáncer, incidencia, mortalidad, registros poblacionales

## Abstract

**Objective:**

To describe cancer incidence and mortality during the 2008-2012 period in the District of Barranquilla.

**Methods:**

Cancer incident cases were collected, analyzed and processed by the Barranquilla Population Cancer Registry during the study period. Population structure was obtained from the *Departamento Administrativo Nacional de Estadìsticas* (DANE) and mortality from the *Sistema de Información de Cáncer en Colombia*. The total and specific crude and specific incidence rates and mortality by age and gender were estimated, as well as by-age standardized incidence rates. Statistical analysis were performed with SPSS V24 and Canreg5.

**Results:**

8,182 cases of cancer were identified, excluding non-melanoma skin cancer (62.8% in women). 83.0% of the tumors had histological verification and only 5.2% were DCO. The adjusted incidence rate for all tumors was 116.5 per 100,000 in men and 155.4 per 100,000 in women. The most frequent locations were prostate and trachea-bronchi-lung in men, while in women, breast and cervix occupied the first places. Breast and prostate had the highest mortality rates in women and men, respectively.

**Conclusion:**

Specific behavior of cancer incidence and mortality in Barranquilla has important increases for the main types of tumors (breast and prostate) when compared to the country and other population registries. To provide data is key to showing a representative behavior of the Colombian Caribbean.

## Resumen

**Objetivo:**

Describir el comportamiento de la incidencia y mortalidad del cáncer durante el período 2008-2012, en el Distrito de Barranquilla, Colombia.

**Métodos:**

La información fue obtenida del Registro Poblacional de Cáncer de Barranquilla y el DANE. Se analizaron casos incidentes y muertes por cáncer en residentes de Barranquilla, desde enero 1 de 2008 hasta diciembre 31 de 2012. Los casos informados corresponden a tumores malignos primarios invasivos en todas las localizaciones, excepto carcinomas de células escamosas y células basales en piel. Se verificó la consistencia interna de los datos y se aplicaron indicadores de calidad sugeridos por la IARC. La población en riesgo se obtuvo de proyecciones poblacionales (1985-2020, DANE). Se estimaron tasas específicas por sexo y edad (grupos quinquenales) y se estandarizaron por método directo utilizando como referencia población mundial.

**Resultados:**

Se identificaron 8,182 casos de cáncer, (62.8% en mujeres). 83.0% tuvieron verificación histológica y 5.2% fueron capturados sólo por certificado de defunción. La tasa global de incidencia de cáncer por 100,000 personas /año ajustada por edad fue 116.5 en hombres y 155.4 en mujeres. Las localizaciones más frecuentes de neoplasias malignas fueron: próstata y pulmón en hombres, y mama y cérvix para mujeres. Las mayores tasas de mortalidad se presentaron por los tumores de mama en mujeres y próstata en hombres.

**Conclusión:**

la incidencia y mortalidad por cáncer en Barranquilla presentan aumentos importantes en los principales tumores (mama y próstata) con respecto a otras regiones de Colombia. Los datos aportados pueden considerarse representativos del comportamiento epidemiológico del cáncer en el caribe colombiano.

## Introduction

Cancer is a public health problem. In 2012, there were near of 14 million incident cases worldwide, and the expectation is unfavorable since this number is expected to increase to almost 24 million by 2030 ^1^. Cancer is responsible for 17% of the deaths in the world, and about a third of them are due to five potentially modifiable risk factors: high body mass index, low intake of fruits and vegetables, cigarette smoking, sedentary lifestyle and consumption of alcohol ^2^, which indicates that cancer is largely preventable.

In Colombia, cancer is the third cause of mortality ^3^. For the 2002-2006 period, the age-standardized incidence rate for all cancers (with the exception of skin) was 196.9 / 100,000 in women and 186.6 in men ^4^, similar to international rates, noting that close to 56% of new cases and about 70% of deaths from cancer occur in medium and low-income countries ^5^.

Some researchers point out that cancer mortality rates can be taken as an indicator of health care´s quality, due to the disease’s high probability of being prevented or treated in a timely manner ^6^. Therefore, it is necessary to determine the behavior, not only of the incidence, but also of the mortality, in the different regions of the country ^7^, due to diversity in sociocultural, geographical and genetic characteristics in each area ^4^.

In Colombia, there are only five Population Base Registries endorsed by the *Instituto Nacional de Cancerología* (National Cancer Institute of Colombia, INC): Cali, Bucaramanga, Pasto, Manizales and Barranquilla. Every one of the registers represents the cultural, geographical and environmental differences of the zones that each covers; nevertheless, the city of Barranquilla is the only one that does not have direct environmental influences and Andean customs, such as those of the interior of the country, palpable in the other Registries; being a coastal city, with a tropical climate and with characteristic that include genetic, social and cultural elements, a product of the miscegenation marked since the Spanish colonization and having been in previous centuries the recipient of European and Middle Eastern migrants, it is necessary to have a population register of cancer and analyze the behavior of cancer in the city within the national scenario, which gave rise to the Barranquilla Population Cancer Registry (BPCR), the result of a strategic alliance between the INC and the *Universidad del Norte* to consolidate incidents in the population resident in the District of Barranquilla since January 1^st^, 2008, and have quality information, which is necessary for cancer control.

The objective of present study is to describe cancer incidence and mortality in Barranquilla for the 2008-2012 period. The BPCR use international standards ^5^ ^,^ ^6^ and the information consolidated by the *Sistema de Información de Cáncer en Colombia* (Cancer Information System in Colombia) to obtain valid information to make timely and efficient decisions regarding the comprehensive approach to cancer in the Colombian Caribbean region.

## Material and Methods

### Type of study

Descriptive Population base 

## Population at risk and area of influence

Barranquilla Population Cancer Registry covers the urban and rural population of the District of Barranquilla, located in the northeastern vertex of the department (province) of *Atlántico*, on the western shore of the Magdalena River, 7.5 km from its mouth in the Atlantic Ocean. Its geographical position is: 10º59'16" north latitude, and 74º47'20" west longitude. The urban area is at a maximum height of 98 meters above sea level to the west and 4 meters above sea level to the east. The city limits to the north with the municipality of Puerto Colombia, to the south with the municipality of Soledad, to the east with the department of Magdalena and the Caribbean Sea, and to the west with the municipalities of Galapa, Puerto Colombia and Tubará. The district of Barranquilla is divided into five localities for administrative and political purposes: *Riomar, Norte-Centro Histórico, Sur Occidente, Metropolitana,* and *Sur Oriente* These localities are subdivided into 611 *manzanas (blocks)* and 188 neighborhoods, approximately. Additionally, the District includes the corregimientos (small towns) of *La Playa* and *Juan Mina* ^8^.

**Figure 1:**
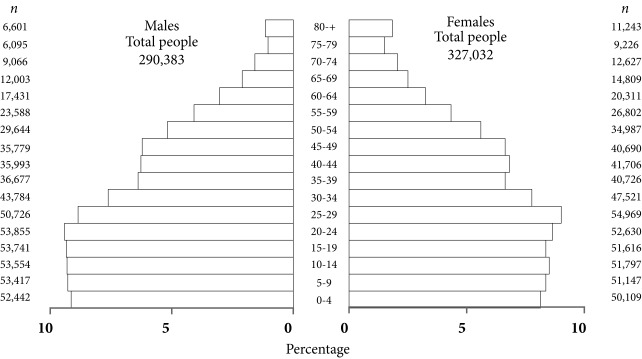
Population structure by age and gender. District of Barranquilla. *Departamento Administrativo Nacional de Estadísticas* (DANE)

The extension of the city is 154 Km^2^, and the climate is dry tropical, with an average temperature of 27.4° C. According to DANE (*Departamento Administrativo Nacional de Estadísticas- National Administrative Department of Statistics)*) projections for the year 2010, Barranquilla had a total of 1,224,000 inhabitants, with a density of 7,945 inhabitants per km^2^. The economy is mainly based on the industrial, port and tourism sectors ^9^. Figure 1 shows the population by gender and age for 2010.

In 2012, the District of Barranquilla had 1,352 private health service providers (807 independent professionals and 463 IPS, which is Healthcare-Providing Institutions or *Instituciones Promotoras de Salud*) and the public network, made up of 48 health institutions, is managed by a private operator. These institutions have more than 70 oncological services (surgical, chemotherapy, radiotherapy, among others) ^10^ that give Barranquilla the status of reference center for the Atlantic Coast and the Caribbean, which is an additional challenge for the BPCR’s aim of identifying cases from city residents that are served by the public and private hospital network. This has been addressed by verifying the data from 74 sources of information (pathology laboratories, imaging centers, clinics and hospitals), each with a different recollection dynamic, and by cross-checking the information from all databases.

The BPCR collects data on malignant tumors (and benign tumors of the central nervous system) in all topographic locations (in skin, only melanoma) and all age groups. The search is active, that is, the BPCR staff periodically visits the sources of information: histopathology laboratories, hospitals and clinics, diagnostic centers, and institutions specialized in oncological care in the city. Likewise, data on mortality, health insurance, and other sociodemographic background is consulted in the official databases of the country, such as the National Registry of the Nation, the "Unique Registry of Affiliates" (*Registro Único de Afiliados,* RUAF) and the "Identification System of Potential Beneficiaries of Social Programs" (*Sistema de Identificación de Potenciales Beneficiarios de Programas Sociales*, SISBEN), among others.

### Barranquilla Population Cancer Registry

It initiated activities in 2008, collecting data from the 2007 cases, which were considered as a pilot test adopting the guidelines suggested by the IARC for population-based records. The BPCR is made up of a multidisciplinary team of 9 people: 3 physicians (one Master in Epidemiology, one Master in Public Health, and one Pathology specialist), one business administrator (coordinator) and 5 technicians (4 information collectors and 1 user) funded by the Universidad del Norte and the INC.

### Collecting and processing information

Barranquilla Population Cancer Registry actively and passively searches for information regarding new cases of cancer.

### Case definition

Every malignant tumor located anywhere, including benign neoplasms of the central nervous system that has been diagnosed in permanent residents of the District of Barranquilla, since January 1^st^ of 2008, regardless of the diagnosis method, including cases identified only by death certificate. Skin cases corresponding to squamous and basal cell carcinomas are excluded (ICD code 10).

Primary cancer is understood as one which originates in a location or tissue that does not correspond to the extension, recurrence or metastasis of another primary tumor ^11^. The most valid basis for diagnosis is the clinical morphology (histopathological, aspiration cytology, flow cytometry, imaging, endoscopy) and death certificate only (DCO).

The main information sources of the registry are: pathology and hematology laboratories, hospital discharges, imaging and early detection centers, oncology centers, medical specialists, and individual death certificates. To confirm if a subject is a resident of Barranquilla, or has a high probability of being a resident, the identification document number is used to validate residence; this is entered on the *Registraduría Nacional* (National Register) website (http://www.registraduria.gov.co/), and the subject that appears with an assigned voting location is considered as a habitual resident of said place; the information is then cross-checked with the official national database of beneficiaries of social programs (https://www.sisben.gov.co/atencion-al-ciudadano/Paginas/consulta-del-puntaje.aspx; http://roble.barranquilla.gov.co:8888/SisbenIII/) and healthcare (http://ruafsvr2.sispro.gov.co/; http://www.adres.gov.co/BDUA/) which include addresses. Likewise, through specific projects, cases are selected that are analyzed in depth regarding sociodemographic and clinical variables.

The cases obtained from hospital discharges on one hand and DCO on the other hand, are determined after a process of review of clinical histories to verify their diagnosis. In the absence of clinical information, cases are labeled as "identified only by DCO".

### Classification and codification of cases

The BPCR collects patient variables (identification number, name, gender, and age / date of birth) and tumor variables (date of incidence, valid basis of diagnosis, topography, morphology, behavior and source of information). The coding is carried out by personnel trained in the application of ICD-O-310 guidelines. The information was initially processed in an electronic sheet and was migrated to Canreg5 in 2016. The coding of the cases is carried out following the Third Edition of the International Classification of Oncological Diseases (ICD-O-3) ^12^ and the rules for multiple primary tumors of the International Agency for Research on Cancer (IARC) ^13^. For the definition of the incidence date, the recommendations of the European Network of Cancer Registries (ENCR) ^14^ are considered. The database is reviewed with IARCtools® and LinkPlus® to identify possible errors and duplications, and to verify the internal consistency between variables ^6^. Cases with inconsistencies are reviewed in the sources of information and adjustments are made.

### Quality of the information

BPCR researchers, supported by the INC and the population registries of Cali, Bucaramanga, Manizales, and Pasto, permanently train the people participating in the RPCB in techniques and standards to collect, process and analyze information, while being aware of the fact that the quality of the data depends on the information obtained from the sources and of the mission processes of the BPCR. In addition, indicators suggested by the IARC are used to evaluate the quality of the BPCR: percentage of cases with microscopic verification, percentage of cases registered only by DCO, percentage of cases with unknown primary location, proportion of cases with unknown age at the time of diagnosis, mortality / incidence ratio, and percentage of cases with unknown diagnosis basis. Regarding confidentiality, the BPCR adopts the standards of the IARC, considering the purpose of collecting, processing and analyzing the information as epidemiological ^15^.

### Estimations of incidence

Every new case registered in residents of Barranquilla between January 1^st^ of 2008 and December 31^st^ of 2012 was considered. The population at risk was calculated using the 1985-2020 projections prepared by DANE. The specific rates were estimated by gender and age (five-year groups) and standardized using the direct method when using the world population (WHO) as a reference. The relative frequencies of incident cases were estimated by specific locations. Incidence and mortality data are presented grouped in ICD-10 codes for comparability purposes, following the methodology used by the IARC ^16^.

### Estimations of mortality

All deaths occurred during the same period, were included with the ICD-10 codes corresponding to malignant neoplasms, including DCOs and deaths occurred in the observation period. It was based on the information consolidated in the Cancer Information System in Colombia ^17^, which uses as population at risk the 1985-2020 projections done by DANE. Mortality was also adjusted by the direct method using the same reference population used in the standardization of incidence.

## Results

### Global quality indicators

The percentage of histological verification for all locations was of 80.0% in men and of 85.9% in women; the five main locations by gender showed that breast, cervix and thyroid (in women), and leukemia (in men) had percentages higher than 90%. On the other hand, the percentage of registrations by DCO represented 3.9% in women and 6.5% in men and had values ​​lower than 5.0% for prostate and leukemia (men) and breast, cervix and thyroid (women). Regarding the Mortality-Incidence ratio, for men it had a value of 0.5 and for women of 0.7. It is striking that, Leukemia, Lung and Stomach in men, and Lung in women, had values ​​higher than 1. On the other hand, there were no cases without information in the gender and diagnostic basis variables. Table 1 shows the quality indicators for the locations with the highest incidence by gender.

**Table 1:** Quality indexes by high incidence cancer location and gender. BPCR, 2008-2012

Location	Male				Female			
	n	%DCO	%MV	MI	n	%DCO	%MV	MI
Breast					2,094	1.0	93.0	0.2
Cervix uteri					747	2.1	94.6	0.4
Thyroid					176	0.0	91.5	0.1
Prostate	1,078	2.6	85.6	0.4				
Leukaemia	156	4.5	96.2	2.4				
Stomach	113	9.7	68.1	1.2				
Lung	265	13.2	64.9	1.4	159	18.9	61.0	1.5
Colon	137	10.2	73.0	0.6	175	7.4	74.3	0.7
Total	3,042	6.5	80.0	0.5	5,140	3.9	85.9	0.7

DCO: only via death certificate

MV: microscopic verification.

MI: Mortality to incidence ratio

### Incidence and mortality due to every cancer (all locations)

During the period of study, 8,182 new cases were registered, 62.8% of which corresponded to women. The average age at diagnosis was 56.1 years old for women and 61.9 for men, and 2.3% of cases occurred in the pediatric population (younger than 15 years old). The Age-Standardized Incidence Rate per 100,000 people-year for all primary locations, including melanoma and excluding the rest of skin tumors, was 116.5 in men and 155.4 in women and the female / male incidence ratio was 1.3 (Table 2). Regarding mortality, it was higher in men: 82.4 compared to 75.9 deaths per 100,000 people-years and the ratio of female to male mortality was 0.92 (Table 3).

**Table 2:** Cancer incidence by location and gender. Barranquilla, 2008-2012

Location	Males			Females			ICD-10 Code
	n	CR	ASIR	n	CR	ASIR	
Oral cavity and pharynx	125	4.3	4.6	74	2.4	2.2	C00-C14
Esophagus	34	1.1	1.3	22	0.7	0.6	C15
Stomach	117	4.1	4.4	96	3.1	2.8	C16
Small intestine	10	0.3	0.3	10	0.3	0.2	C17
Colon and rectum	253	8.8	9.6	337	11.0	9.8	C18-C20
Anus	12	0.4	0.4	48	1.5	0.9	C21
Liver and bile ducts	51	1.9	2.1	56	1.9	1.8	C22
Gallbladder	19	0.7	0.8	34	1.3	1.9	C23-C24
Pancreas	41	1.1	1.2	66	1.2	1.0	C25
Nose. ear and paranasal sinus	10	0.3	0.4	9	0.2	3.2	C30-C31
Larynx	103	3.6	3.9	18	0.6	3.5	C32
Lung	268	9.4	10.4	159	5.4	4.8	C33-C34
Other thoracic organs	19	0.7	0.7	19	0.6	0.5	C37-C38
Bones and articulations	38	1.2	1.2	33	1.0	1.0	C40-C41
Melanoma of the skin	10	0.3	0.4	13	0.4	0.4	C43
Conjunctive and soft tissue	71	2.4	2.6	85	2.7	2.6	C47-C49
Breast				2,148	70.0	65.7	C50
Vulva				19	0.7	0.6	C51
Vagina				38	1.2	1.1	C52
Cervix uter				870	28.7	26.6	C53
Body of the uterus				100	3.1	2.9	C54
Ovary				143	4.7	4.4	C56
Other female organs Not Specified				8	0.3	0.3	C57
Penis	40	1.4	1.6				C60
Prostate	1,104	37.5	4.3				C61
Testícle	11	0.4	0.4				C62
Kidney	62	2.0	2.3	53	1.7	1.6	C64
Bladder	66	2.3	2.6	33	1.0	0.9	C67
Eyes and anexes	26	1.1	1.1	14	0.5	0.5	C69
Brain, CNS	107	3.7	3.9	100	3.2	3.0	C70-C72
Thyroid	31	1.0	1.1	182	5.8	5.2	C73
Other endocrine glands	10	0.3	0.3	9	0.2	0.2	C75
Hodgkin Lynphoma	35	1.1	1.1	26	0.9	0.8	C81
Non Hodgkin Lynphoma	107	3.8	4.0	9	2.6	2.4	C82-C85,C96
Multiple myeloma	17	0.6	0.6	18	0.6	0.5	C90
Lymphoid leukaemia	52	1.8	1.9	27	1.5	1.5	C91
Myeloid leukaemia	69	2.2	2.3	27	1.9	1.8	C92-C94
Leukaemia unspecified	37	1.4	1.5	27	0.9	0.8	C95
Other and unspecified	90	3.0	3.1	79	2.5	2.2	C26,C39,C48,C76,C80
All locations	3,063	105.9	116.5	5,133	168.0	155.4	C00-C96

CR: Crude rate per 100,000 people-year,

ASIR: Age-standardized Incidence Rate (Segi world population) per 100,000 people-year

**Table 3:** Annual average mortality due to cancer by location and gender. Barranquilla, 2008-2012

Location	Males			Females			ICD-10 Code
	n	CR	ASIR	n	CR	ASIR
Oral cavity and pharynx	10	1.7	1.9	6	0.9	0.8	C00-C14
Esophagus	7	1.1	1.3	3	0.4	0.3	C15
Stomach	27	4.6	5.1	24	4.0	3.3	C16
Colon and rectum	33	5.7	6.0	49	7.9	6.7	C18-C20
Liver and bile ducts	18	3.1	3.4	23	3.7	3.1	C22
Pancreas	16	2.8	3.1	22	3.5	2.9	C25
Lung	75	13.0	14.3	49	8.1	7.1	C33-C34
Melanoma of the skin	2	0.3	0.4	2	0.2	0.2	C43
Breast				108	17.6	15.7	C50
Cervix uter				66	10.8	9.8	C53
Body of the uterus				6	0.9	2.9	C54
Ovary				21	3.4	3.1	C56
Prostate	95	16.6	17.4				C61
Bladder	7	1.3	1.3	5	0.8	0.6	C67
Lymphomas	27	4.7	5.1	24	3.9	3.5	C81-C90, C96
Leukemia	34	5.9	6.1	28	4.6	4.4	C91-C95
Thyroid	6	1.0	0.2	35	5.8	0.5	C73
Other and unspecified	24	4.1	4.4	32	5.3	4.5	C26, C39,C48,C76,C80
All locations	442	76.9	82.4	527	86.1	75.9	C00-C96

CR: Crude rate per 100,000 people-year,

ASIR: Age-standardized Incidence Rate (Segi world population) per 100,000 people-year

### Incidence and mortality by type of cancer

**Figure 2:**
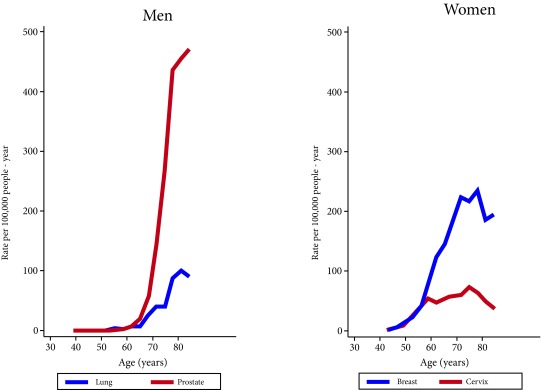
Specific incidence rates by age in women and men, first two locations. (Rates per 100,000 p-y). Barranquilla, 2008-2012

The five locations with the highest incidence in men were: prostate (43.0), trachea, bronchus and lung (10.4), colon and rectum (9.6), oral cavity (4.6) and stomach (4.4), corresponding to 61.3% of all the types of cancer. In women, the five most recurrent types of cancer represent 72.0% of all types of cancer, and were: breast (65.7), cervix (26.6), colon and rectum (9.8), thyroid (5.2), and trachea, bronchi and lung (4.8). In terms of mortality, tumors in the lung, breast, prostate, colon and rectum and cervix represent 49.0% of all tumors, and the highest mortality rates standardized by age per 100,000 people / year were: prostate (17.4), lung (14.3), leukemia (6.1) and stomach and lymphomas (5.1 each) in men, and breast (15.7), cervix (9.8), lung (7.1), colon and rectum (6.7), and leukemia (4.4) in women. Tables 2 and 3 show the incidence and mortality rates according to specific locations by gender, and Figure 2 shows the incidence rate by age for the two main types of cancer in women (breast and cervix) and men.

## Discussion

Regarding the quality criteria, the BPCR was found to be compliant with the requirements defined by the IARC ^6^: microscopic verification of at least 80% of the cases and less than 10% of the cases registered by DCO, and the same figure for tumors with an unknown or a poorly defined primary location. It is possible that the M:I >1 ratio for some tumors may indicate the need to strengthen the active search for incident cases, but it could also indicate an excessive registration of lung, leukemia and stomach cancer as causes of death in the DCOs.

With respect to the results of the analysis of the cases registered by the BPCR during the 2008-2012 period, the standardized rate in women (155.4/100,000) was higher than that of men (116.5/100,000), as it has occurred in other places, such as Guayaquil, Ecuador, whose Population Registry found rates of 110.0/100,000 and 146.0/100,000 for men and women, respectively, during the 2003-2006 period ^18^, and in Khartoum, Sudan ^19^, which also had larger rates for women: 124.3/100,000 and 90.8/100,000, during a period similar to the one reported in this article: 2009-2012.

On the other hand, more than 80% of the cases had pathological confirmation, and the percentages of cases identified only by DCO were 6.5% in men and 3.9% in women, these were values ​​lower than the maximum suggested by the IARC ^20^. The cancers with higher incidence in women were: breast, cervix, colon-rectum, thyroid, and trachea-bronchi-lung. For men, the malignant neoplasms of higher incidence were: prostate, trachea-bronchi-lung, colon-rectum, oral cavity, and stomach. The standardized rate for all cancers, excluding non-melanoma skin cancer, was 116.5 per 100,000 people-years in men and 155.4 per 100,000 person-years in women.

Cervical tumors represented a significant percentage, which can be attributed, to a large extent, to the early detection programs in the country ^20^, which have contributed to the inclusion of these cancers, along with breast cancer, as an epidemiological surveillance object ^21^. It is worth noting the high incidence of breast cancer (65.7/100,000), higher than those reported for different periods (2003-2007) by Manizales (33 / 100,000) ^22^, Bucaramanga (41.9/100,000) ^23^, and Cali (48.0/100,000) ^24^. This data is related to mortality from this tumor, since along with Armenia, Cali, and Bucaramanga, Barranquilla has mortality rates which are higher than the national average: 10.5 ^25^, and although mortality has been determined chronologically before the incidence, it could be an indicator associated with the number of cases captured by the BPCR, which is supported by the number of cases of breast cancer estimated for the department of Atlántico by the INC ^4^: 481 per year, compared to an annual average of 430 captured by the BPCR. Likewise, the percentage of DCO is low for breast cancer, and having carried out an exhaustive review of the residence for these tumors ^26^, this information is validated to a great extent, although for future studies the effect of sociocultural factors (use of screening, self-care, etc.) environmental (climate, topography, altitude) ^25^ and the composition of the population, a product not only of miscegenation, but also of groups that migrated to the area since the nineteenth century (Arabs, Germans and English, etc.) ^27^.

In the case of men, prostate cancer presented a rate of 43 cases per 100,000 men-years, a value that could be considered as intermediate when compared with Bucaramanga (50.5) and Manizales (32.7). In this regard, it is important to mention that, in Colombia, the *Ministerio de Salud y Protecciòn Social* (Ministry of Health and Social Protection) and the *Sociedad Colombiana de Urologìa* (Colombian Society of Urology) recommend early detection in men over 50 years of age or under 50 years of age if risk factors are present ^28^, due to scientific evidence showing better results for screening in this groups.

Regarding mortality, important differences have been found in some departments of Colombia, such as the case of *Atlántico*, where the District of Barranquilla is located, where mortality rates for cancer were higher than the national average ^4^; for the 2007-2011 period, the mortality rate standardized by age for breast cancer per 100,000 inhabitants was 9.5 in the country, while for the department of *Atlántico* this indicator had a value of 12.0, only exceeded by *Valle del Cauca* with 12.3, while for prostate at national level the value was of 10.5 per 100,000 inhabitants, and for the Department it was of 14.6, occupying the second place at the national level ^4^.

This study finds that the rates for breast and prostate cancers are the highest: 15.7 and 17.4 per 100,000 inhabitants in each case, as stated by the *Análisis de Situación de Salud del Distrito de Barranquilla* (Health Situation Analysis of the District of Barranquilla) ^10^, which also indicates that the mortality rate of breast cancer has progressively increased by 2.43 points from 2004 to 2014, while in the same period, prostate cancer’s has fallen 5.36 points.

It is noteworthy that, this study is the first to take data from the *Sistema de Información de Cáncer en Colombia* to analyze mortality. This tool is available to the general public as of 2017 and is the result of the efforts of the INC and the Cancer Registries of Colombia. Taking this source and not the DCOs of DANE "directly", is an interesting challenge that allows us to glimpse the scope and potential of the information system as an important resource for decision making in the country. Breast cancer was found to be the leading cause of death among women, and in men, cancer mortality was attributed mainly to prostate cancer.

## Conclusions

The information obtained by the BPCR is reliable, in accordance with the standards required by IARC, and constitutes an important contribution to the *National Information System of Cancer.* The epidemiological behavior of cancer in Barranquilla has variations with regards to what was found and reported during the previous years in the country, although the risk of developing cancer or dying due to it is considered intermediate when compared with the figures reported by other registries.

The estimates made for the 2008-2012 five-year period will serve as a baseline for the construction of future trends. The information generated by the BPCR provides a valuable contribution to the construction of reliable epidemiological information for the country, specifically in a representative city of the north coast of Colombia, so that its sustainability must be guaranteed and every day its objectives and strategies must be improved for both medium and long term.
