# Supplementary material for: Cancer incidence and mortality in Barranquilla, Colombia. 2008-2012
Source: Colomb Med (Cali). 2018 Mar 30;49(1):55–62. doi: 10.25100/cm.v49i1.3627 (PMC6018819; doi:10.25100/cm.v49i1.3627)
Supplement: Related texts

# Research article: Incidencia y mortalidad por cáncer en Barranquilla, Colombia. 2008-2012

## Introducción

El cáncer es un problema de salud pública. para el año 2012 hubo alrededor de 14 millones de casos incidentes en todo el mundo, y la expectativa es desfavorable, se espera que esta cifra aumente a casi 24 millones para el año 2030 ^1^. El cáncer es responsable de 17% de las muertes en el mundo, y alrededor de un tercio son debidas a cinco factores de riesgo potencialmente modificables: índice de masa corporal elevado, baja ingesta de frutas y verduras, consumo de cigarrillo, sedentarismo y consumo de alcohol ^2^, lo cual indica que el cáncer es prevenible en gran manera.

 En Colombia, el cáncer es la tercera causa de mortalidad ^3^. Para el período 2002-2006 la tasa de incidencia ajustada por edad para todos los cánceres (excepto piel) fue de 196.9/100,000 en mujeres y 186.6 en hombres ^4^, cifras similares al escenario internacional, con la salvedad que en países de medianos y bajos ingresos, ocurren cerca del 56% de los casos nuevos de cáncer y alrededor de 70% de las muertes por cáncer ^5^.

Algunos investigadores señalan que la mortalidad por cáncer puede ser tomada como un indicador de calidad de atención en salud, por su alta probabilidad de ser prevenible o tratado oportunamente ^6^. Por ello, resulta necesario determinar el comportamiento no solamente de la incidencia sino también de la mortalidad en las diferentes regiones del país ^7^, debido la diversidad en las características socioculturales, geográficas y genéticas en cada zona geográfica ^4^. 

En Colombia solamente existen cinco Registros de cáncer de Base Poblacional avalados por el Instituto Nacional de Cancerología de Colombia (INC): Cali, Bucaramanga, Pasto, Manizales y Barranquilla. Cada uno de los registros representa las diferencias culturales, geográficas y ambientales propias de la zona andina; sin embargo, la ciudad de Barranquilla al ser una ciudad costera, de clima tropical y con elementos genéticos, sociales y culturales característicos, producto del mestizaje marcado desde la colonización española y de haber sido en siglos anteriores receptora de migrantes europeos y de oriente medio, determina un tipo de población diferente, esto hace necesario un registro poblacional de cáncer en esta zona del país para poder analizar el comportamiento del cáncer en la ciudad y dentro del panorama nacional. Esto dio origen al Registro Poblacional de Cáncer de Barranquilla (RPCB), resultado de una alianza estratégica entre el INC y la Universidad del Norte para consolidar casos incidentes en la población residente en el Distrito de Barranquilla a partir del primero de enero de 2008 y contar con información de calidad, necesaria para el control del cáncer.

El objetivo del presente estudio fue describir la incidencia y mortalidad por cáncer en Barranquilla para el período 2008-2012. El RPCB utilizó estándares internacionales^5,6^ y la información consolidada por el Sistema de Información de Cáncer en Colombia para obtener información válida que permita tomar decisiones oportunas y eficientes en relación con el abordaje integral del cáncer en la región Caribe colombiana.

## Materiales y Métodos

### Tipo de estudio 

Descriptivo de base poblacional.

### Población en riesgo y área de influencia

El RPCB cubre la población urbana y rural del Distrito de Barranquilla, ubicado en el vértice nororiental del departamento del Atlántico, sobre la orilla occidental del río Magdalena, a 7.5 km de su desembocadura en el Océano Atlántico. Su posición geográfica es: latitud 10º59’16” norte, y longitud 74º47’20” occidente. El área urbana se encuentra a una altura máxima sobre el nivel del mar de 98 metros al occidente y 4 metros al oriente. La ciudad limita al norte con el municipio de Puerto Colombia, al sur con el municipio de Soledad, al oriente con el departamento del Magdalena y el Mar Caribe, y al occidente con los municipios de Galapa, Puerto Colombia y Tubará. El distrito de Barranquilla está dividido administrativa y políticamente en cinco localidades: Riomar, Norte-Centro Histórico, Sur Occidente, Metropolitana y Sur Oriente Estas localidades se subdividen en 611 manzanas y 188 barrios, aproximadamente. Adicionalmente, el Distrito comprende los corregimientos de La Playa y Juan Mina ^8^.

La extensión de la ciudad es de 154 Km^2^ y el clima es de tipo tropical seco, con una temperatura promedio de 27.4° C. Según datos de Proyección del DANE, para el año 2010, Barranquilla tenía un total de 1,224,000 habitantes, con una densidad de 7,945 habitantes por Km^2^. La economía está sustentada principalmente en el sector industrial, portuario y turístico ^9^. La Figura 1 muestra la estructura de la población por sexo y edad para el año 2010.

**Figura 1:** Distrito de Barranquilla. Estructura poblacional por edad y sexo en el año 2010.Fuente: Proyecciones censales DANE 2010

En el año 2012 el Distrito de Barranquilla contaba con 1,352 prestadores de Servicios de Salud privados (807 profesionales independientes y 463 IPS) y la red pública, conformada por 48 instituciones de salud, es administrada por un operador privado. Estas instituciones poseen más de 70 servicios oncológicos (quirúrgicos, quimioterapia, radioterapia, entre otros) ^10^ que le confieren a Barranquilla el carácter de centro de referencia de la Costa Atlántica y el Caribe, lo cual es un reto adicional para el RPCB, para la identificación de casos residentes en la ciudad que son atendidos en la red hospitalaria pública y privada. Esto se ha afrontado, mediante la verificación de datos en las 74 fuentes de información (laboratorios de patología, centros de imagenología, clínicas y hospitales) cada una con una dinámica de recolección diferente, y al cruce de información con las bases de datos.

El RPCB reúne datos sobre tumores malignos (y los benignos del sistema nervioso central) en todas las localizaciones topográficas (en piel solamente melanoma) y de todos los grupos de edad. La búsqueda de casos es activa, es decir, el personal del RPCB visita periódicamente las fuentes de información: laboratorios de histopatología, hospitales y clínicas, centros de diagnóstico e instituciones especializadas en atención oncológica en la ciudad. Así mismo, se consultan datos sobre mortalidad, de aseguramiento en salud, y otros antecedentes sociodemográficos en las bases de datos oficiales del país como la de la Registraduría General de la Nación, la del “Registro Único de Afiliados” (RUAF) y la del “Sistema de Identificación de Potenciales Beneficiarios de Programas Sociales” (SISBEN), entre otras. 

### El Registro Poblacional de Cáncer de Barranquilla 

Inició actividades en el año 2008, recolectando datos sobre los casos de 2007 que se consideraron como prueba piloto adoptando las guías sugeridas por la IARC para registros de base poblacional. el RPCB está constituido por un equipo multidisciplinario de 9 personas: 3 médicos (un Magíster en Epidemiología, un Magíster en Salud Pública y una especialista en Patología), una administradora de empresas (coordinadora) y 5 técnicos (4 recolectores de información y 1 digitadora) financiado por la Universidad del Norte y el Instituto Nacional de Cancerología de Colombia. 

### Recopilación y procesamiento de la información

El RPCB lleva a cabo búsqueda activa y pasiva de información de casos nuevos de cáncer. 

### Definición de caso

Todos los tumores malignos localizados en cualquier sitio, incluyendo neoplasias benignas del sistema nervioso central, que hayan sido diagnosticados en residentes permanentes en el Distrito de Barranquilla, a partir del primero de enero de 2008, independientemente del método de diagnóstico, incluidos los casos identificados únicamente por certificado de defunción. Se excluyen los casos de piel correspondientes a escamocelulares y basocelulares (código CIE 10).

Como cáncer primario, se entiende aquel que se origina en un sitio o tejido que no corresponde a la extensión, recurrencia o metástasis de otro tumor primario ^11^. La base más válida de diagnóstico es la morfología (histopatológica, citología aspirativa, citometría de flujo), imágenes, endoscopia, clínica y única evidencia de cáncer certificado de función. 

Las principales fuentes de información del registro son: laboratorios de patología y hematología, egresos hospitalarios, centros de imagenología y de detección precoz, centros oncológicos, médicos especialistas, y los certificados individuales de defunción. Para confirmar si un sujeto es residente en Barranquilla, o tiene una alta probabilidad de serlo, se emplea el número del documento de identificación para validar residencia; este es ingresado en la página de la Registraduría Nacional (http://www.registraduria.gov.co/), y se considera como residente habitual aquel sujeto que aparezca con lugar de votación asignado; la información se contrasta con las bases oficiales del país para beneficios (https://www.sisben.gov.co/atencion-al-ciudadano/Paginas/consulta-del-puntaje.aspx; http://roble.barranquilla.gov.co:8888/SisbenIII/) y aseguramiento (http://ruafsvr2.sispro.gov.co/; http://www.adres.gov.co/BDUA/) en las que se hace mención a residencia. Así mismo, a través de proyectos específicos, se seleccionan casos que son analizados a profundidad con respecto a las variables sociodemográficas y clínicas. 

Los casos obtenidos de egresos hospitalarios, por una parte y DCO por otra parte, se determinan luego de un proceso de revisión de historias clínicas para verificar su diagnóstico. En ausencia de información clínica, los casos son etiquetados como “identificados solo por DCO”. 

#### Clasificación y codificación de casos

El RPCB recolecta variables del paciente (número de identificación, nombre, sexo y edad/fecha de nacimiento) y del tumor (fecha de incidencia, base válida de diagnóstico, topografía, morfología, comportamiento y fuente de información). La codificación es llevada a cabo por personal entrenado en la aplicación de las pautas ICD-O-310. La información es procesada inicialmente en una hoja electrónica y en el año 2016 fue migrada a Canreg5. La codificación de los casos se lleva a cabo teniendo en cuenta la Tercera Edición de la Clasificación Internacional de Enfermedades Oncológicas (CIE-O-3) ^12^ y las reglas para tumores primarios múltiples de la Agencia Internacional para la Investigación en Cáncer (IARC) ^13^. Para la definición de fecha de incidencia, se tienen en cuenta las recomendaciones de la Red Europea de Registros del Cáncer (ENCR) ^14^. La base de datos es revisada con IARCtools^®^ y LinkPlus^®^ para identificar posibles errores y duplicidades y verificar la consistencia interna entre las variables ^6^. Los casos con inconsistencias son revisados en las fuentes de información y se les hacen los ajustes pertinentes. 

#### Calidad de la información

A través de un entrenamiento y capacitaciones permanentes por parte de los investigadores del RPCB y con el apoyo del Instituto Nacional de Cancerología y los registros poblacionales de Cali, Bucaramanga, Manizales y Pasto, las personas que participan del RPCB son formadas en técnicas y normas para recolectar, procesar y analizar la información, conscientes que la calidad de los datos depende de la información obtenida en las fuentes y de los procesos misionales del RPCB. Además, indicadores sugeridos por la IARC, son empleados para evaluar la calidad del RPCB: porcentaje de casos con verificación microscópica, porcentaje de casos registrados solo por DCO, porcentaje de casos con localización primaria desconocida, proporción de casos con edad al momento del diagnóstico desconocida, razón Mortalidad/Incidencia y porcentaje de casos con base diagnóstica desconocida. Con respecto a la confidencialidad de la información, el RPCB adopta los estándares de la International Agency for Cancer Research (IARC), considerando que la finalidad de recolectar, procesar y analizar la información es de tipo epidemiológica ^15^. 

#### Estimaciones de incidencia

Se consideraron todos los casos nuevos ocurridos en personas residentes en Barranquilla entre el 1 de enero de 2008 y el 31 de diciembre de 2012. Como población en riesgo se utilizaron las proyecciones de población de 1985-2020 elaboradas por el DANE sobre la base de conciliaciones censales. Las tasas específicas se estimaron por sexo y edad (grupos quinquenales), y se estandarizaron mediante el método directo al usar la población mundial (OMS) como referencia. Las frecuencias relativas de casos incidentes se estimaron por localizaciones específicas. Los datos de incidencia y mortalidad se presentan agrupados en los códigos de la CIE-10 con fines de comparabilidad, siguiendo la metodología utilizada por la IARC ^16^.

#### Estimaciones de mortalidad

Se incluyeron todas las muertes ocurridas durante el mismo período con los códigos CIE-10 correspondientes a neoplasias malignas, incluyendo los DCO y las muertes ocurridas en el período de observación, a partir de la información consolidada en el Sistema de Información de Cáncer en Colombia ^17^, el cual utiliza como población en riesgo las proyecciones de 1985-2020 elaboradas por el DANE. La mortalidad también fue ajustada por el método directo usando la misma población de referencia empleada en la estandarización de la incidencia.

## Resultados

### Indicadores de calidad global

El porcentaje de verificación histológica para todas las ubicaciones fue de 80.0% en hombres y 85.9% en mujeres; las cinco principales localizaciones por sexo mostraron que mama, cérvix y tiroides (en mujeres) y leucemia (en hombres), tuvieron porcentajes superiores a 90%. Por su parte, el porcentaje de registros por DCO representó el 3.9% en mujeres y 6.5% en hombres y tuvo valores inferiores a 5.0% en próstata y leucemia (hombres) y mama, cérvix y tiroides (mujeres). En cuanto a la relación Mortalidad-Incidencia, para los hombres fue de 0.5 y para las mujeres 0.7. Llama la atención que, en el caso de los hombres Leucemia, Pulmón y Estómago en hombres, y Pulmón en mujeres, tuvieron valores superiores a 1. Por otro lado, no se registraron casos sin información en las variables sexo y base diagnóstica. En la Tabla 1 se pueden observar los indicadores de calidad para las localizaciones de mayor incidencia por sexo.

**Tabla 1:** Indicadores de calidad, por localizaciones topográficas de alta incidencia por sexo. RPCB 2008-2012.

Localización	Hombres				Mujeres			
	n	%DCO	%VM	M:I	n	%DCO	%VM	M:I
Mama					2,094	1.0	93.0	0.2
Cérvix					747	2.1	94.6	0.4
Tiroides					176	0	91.5	0.1
Próstata	1,078	2.6	85.6	0.4				
Leucemia	156	4.5	96.2	2.4				
Estómago	113	9.7	68.1	1.2				
Pulmón	265	13.2	64.9	1.4	159	18.9	61.0	1.5
Colon	137	10.2	73.0	0.6	175	7.4	74.3	0.7
Total	3,042	6.5	80.0	0.5	5,140	3.9	85.9	0.7

DCO (Por su sigla en inglés): Unica evidencia certificado de defunción

VM: Verificación morfológica

MI: razón motalidad/incidencia

### Incidencia y mortalidad debido a todos los cánceres (todas las localizaciones)

Durante el período de estudio, se registraron 8,182 casos nuevos, de los cuales el 62.8% correspondió a mujeres. El promedio de edad al momento del diagnóstico fue de 56.1 años para las mujeres y de 61.9 para los hombres, y el 2.3% de los casos ocurrieron en población pediátrica (menores de 15 años de edad). La Tasa de Incidencia Estandarizada por Edad por 100,000 personas-año para todas las localizaciones primarias, incluido el melanoma y exceptuando el resto de tumores en piel, fue de 116.5 en hombres y 155.4 en mujeres y la razón de incidencia mujer/hombre fue de 1.3 (Tabla 2). Con respecto a la mortalidad, fue más alta en los hombres: 82.4 frente a 75.9 muertes por 100,000 personas-año y la razón de mortalidad mujer/hombre fue de 0.92 (Tabla 3).

**Tabla 2:** Barranquilla, Incidencia de cáncer por localización y sexo. 2008-2012

Localización	Hombres			Mujeres			Codigo ICD-10
	n	TC	TEE	n	TC	TEE	
Cavidad oral	125	4.3	4.6	74	2.4	2.2	C00-C14
Esófago	34	1.1	1.3	22	0.7	0.6	C15
Estómago	117	4.1	4.4	96	3.1	2.8	C16
Intestino Delgado	10	0.3	0.3	10	0.3	0.2	C17
Colon y recto	253	8.8	9.6	337	11.0	9.8	C18-C20
Ano	12	0.4	0.4	48	1.5	0.9	C21
Hígado	51	1.9	2.1	56	1.9	1.8	C22
Vesícula Biliar	19	0.7	0.8	34	1.3	1.9	C23-C24
Páncreas	41	1.1	1.2	66	1.2	1.0	C25
Cavidad nasal	10	0.3	0.4	9	0.2	3.2	C30-C31
Laringe	103	3.6	3.9	18	0.6	3.5	C32
Tráquea, bronquio-pulmón	268	9.4	10.4	159	5.4	4.8	C33-C34
otros organos torácicos	19	0.7	0.7	19	0.6	0.5	C37-C38
Hueso	38	1.2	1.2	33	1.0	1.0	C40-C41
Melanoma	10	0.3	0.4	13	0.4	0.4	C43
Tejidos blandos	71	2.4	2.6	85	2.7	2.6	C47-C49
Mama				2,148	70.0	65.7	C50
Vulva				19	0.7	0.6	C51
Vagina				38	1.2	1.1	C52
Cuello uterino				870	28.7	26.6	C53
Cuerpo uterino				100	3.1	2.9	C54
Ovario				143	4.7	4.4	C56
Otros organos femeninos / no especificado				8	0.3	0.3	C57
Pene	40	1.4	1.6				C60
Próstata	1,104	37.5	43.0				C61
Testículos	11	0.4	0.4				C62
Riñón	62	2.0	2.3	53	1.7	1.6	C64
Vejiga	66	2.3	2.6	33	1.0	0.9	C67
Ojo	26	1.1	1.1	14	0.5	0.5	C69
Cerebro y SNC	107	3.7	3.9	100	3.2	3.0	C70-C72
Tiroides	31	1.0	1.1	182	5.8	5.2	C73
Otras glándulas endocrinas	10	0.3	0.3	9	0.2	0.2	C74
Linfoma de Hodgkin	35	1.1	1.1	26	0.9	0.8	C81
Linfoma No Hodgkin	107	3.8	4.0	9	2.6	2.4	C82-C85,C96
Mieloma Múltiple	17	0.6	0.6	18	0.6	0.5	C90
Leucemias Linfoides	52	1.8	1.9	27	1.5	1.5	C91
Leucemias Mieloides	69	2.2	2.3	27	1.9	1.8	C92-C94
Leucemias NE	37	1.4	1.5	27	0.9	0.8	C95
Otros órganos y no especificado	90	3.0	3.1	79	2.5	2.2	C26,C39,C48,C76,C80
Todas las localizaciones	3,063	105.9	116.5	5,133	168.0	155.4	C00-C96

TC: Tasa Cruda;

TEE: Tasa estandarizada por edad

**Tabla 3:** Tasa de Mortalidad cruda y estandarizada por edad, por 100.000 personas/año, por localización topográfica y género. Barranquilla, 2008-2012

Localización	Hombres			Mujeres			Codigo ICD-10
	n	TC	TEE	n	TC	TEE	
Cavidad oral	10	1.7	1.9	6	0.9	0.8	C00-C14
Esófago	7	1.1	1.3	3	0.4	0.3	C15
Estómago	27	4.6	5.1	24	4.0	3.3	C16
Colon y recto	33	5.7	6.0	49	7.9	6.7	C18-C20
Hígado	18	3.1	3.4	23	3.7	3.1	C22
Páncreas	16	2.8	3.1	22	3.5	2.9	C25
Pulmón	75	13.0	14.3	49	8.1	7.1	C33-C34
Melanoma	2	0.3	0.4	2	0.2	0.2	C43
Mama				108	17.6	15.7	C50
Cuello uterino				66	10.8	9.8	C53
Cuerpo uterino				6	0.9	2.9	C54
Ovario				21	3.4	3.1	C56
Próstata	95	16.6	17.4				C61
Vejiga	7	1.3	1.3	5	0.8	0.6	C67
Linfomas	27	4.7	5.1	24	3.9	3.5	C81-C90, C96
Leucemias	34	5.9	6.1	28	4.6	4.4	C91-C95
Tiroides	6	1.0	0.2	35	5.8	0.5	C73
Otros órganos y no especificados	24	4.1	4.4	32	5.3	4.5	C26, C39,C48,C76,C80
Todas las localizaciones	442	76.9	82.4	527	86.1	75.9	C00-C96

TC: Tasa Cruda;

TEE: Tasa estandarizada por edad

### Incidencia y mortalidad por tipo de cáncer

Las cinco localizaciones con mayor incidencia en hombres fueron: próstata (43.0), tráquea, bronquio y pulmón (10.4), colon y recto (9.6), Cavidad oral (4.6) y Estómago (4.4), correspondientes a 61.3% de todos los tipos de cáncer. En las mujeres, los cinco primeros tipos de cáncer representan el 72.0% de todos los tipos de cáncer, y fueron: mama (65.7), cuello uterino (26.6), colon y recto (9.8), Tiroides (5.2), y tráquea, bronquios y pulmón (4.8). En cuanto a mortalidad, los tumores de pulmón, mama, próstata, colon y recto y cuello uterino representan el 49.0% del total de tumores, y las mayores tasas de mortalidad estandarizadas por edad por 100,000 personas/año fueron: próstata (17.4), pulmón (14.3), leucemias (6.1) y estómago y linfomas (5.1 cada uno) en hombres, y Mama (15.7), Cuello uterino (9.8), Pulmón (7.1), Colon y recto (6.7) y Leucemias (4.4) en mujeres. Las Tablas 2 y 3 muestran las tasas de incidencia y mortalidad según ubicaciones específicas por sexo y las Figura 2 muestran la tasa de incidencia por edad para los dos principales tipos de cáncer en mujeres (mama y cuello uterino) y hombres.

**Figura 2:** Tasa de incidencia específica para hombres y mujeres por edad, primeras dos localizaciones. (Tasa por 100,000 p-a). Barranquilla, 2008-2012.

## Discusión

Se encontró que, con respecto a los criterios de calidad el RPCB cumple con los requisitos definidos por la IARC ^6^: verificación microscópica de al menos el 80% de los casos y menos del 10% de los casos registrados mediante DCO e igual cifra para tumores con sitio primario desconocido o poco definido. Es posible que la razón M:I >1 para algunos tumores puedan indicar la necesidad de fortalecer la búsqueda activa de casos incidentes, pero también podría indicar un registro excesivo del cáncer de pulmón, leucemia y estómago, como causas de muerte en los DCO. 

Con respecto a los resultados del análisis de los casos registrados por el RPCB durante el período 2008-2012, la tasa estandarizada en mujeres (155.4/100,00) fue superior a la de hombres (116.5/100,000), tal como ha ocurrido en otros lugares, como Guayaquil, Ecuador, cuyo Registro Poblacional encontró tasas de 110.0/100,000 y 146.0/100,000 para hombres y mujeres, respectivamente durante el período 2003-2006 ^18^, y en Khartoum, Sudan ^19^^),^ que igualmente tuvo tasas mayores para mujeres: 124.3/100,000 y 90.8/100,000, durante un período similar al reportado en el presente artículo: 2009-2012.

Por otro lado, más del 80% de todos los casos tuvieron confirmación patológica y los porcentajes de casos identificados solo por DCO fue de 6.5% en hombres y 3.9% en mujeres, valores menores al máximo sugerido por la IARC ^20^. Los cánceres con mayor incidencia en mujeres fueron: mama, cuello uterino, colon-recto, tiroides y tráquea-bronquios-pulmón, mientras que en los hombres las neoplasias malignas de mayor incidencia fueron: próstata, tráquea-bronquio-pulmón, colon-recto, cavidad oral y estómago. La tasa estandarizada para todos los cánceres, excluyendo el de piel no melanoma, fue de 116.5 por 100,000 años-persona en hombres y 155.4 por 100,000 años-persona en mujeres. 

Los tumores de cuello uterino representaron un porcentaje importante, lo cual puede ser atribuido en gran medida a los programas para la detección temprana en el país ^20^ y que, han contribuido a la inclusión de estos tipos de cáncer, junto con el de mama, como objeto de vigilancia epidemiológica ^21^. Es de resaltar la alta incidencia de cáncer de mama (65.7/100,000), superior a la reportada en períodos diferentes (2003-2007) por Manizales (33.0/100,000) ^22^ Bucaramanga (41.9/100,000) ^23^ y Cali (48.0/100,000) ^24^. Estos datos se relacionan con la mortalidad por este tumor, ya que junto con Armenia, Cali, y Bucaramanga, Barranquilla presenta tasas de mortalidad superiores al promedio nacional: 10.5 ^25^, y aunque la mortalidad haya sido determinada cronológicamente antes que la incidencia, podría ser un indicador asociado al número de casos captados por el RPCB, lo cual es apoyado por el número de casos de cáncer de mama estimados para el departamento del Atlántico por el INC4: 481 por año, frente a un promedio anual de 430 captados por el RPCB. Así mismo, el %DCO es bajo para mama, y el haber realizado una revisión exhaustiva de la residencia para estos tumores ^26^, valida en gran medida esta información, aunque para futuros estudios se debe considerar el efecto de factores socioculturales (uso de tamizaje, autocuidado, etc.) ambientales (clima, topografía, altitud) ^25^ y su composición poblacional, producto no solo de mestizaje, sino también de grupos que migraron a ella desde el siglo XIX (árabes, alemanes e ingleses, etc.) ^27^. 

En el caso de los hombres, el cáncer prostático presentó una tasa de 43 casos por 100,000 hombres-año, valor que podría considerarse como intermedio al comparar con Bucaramanga (50.5) y Manizales (32.7). Al respecto, es importante mencionar que, en Colombia, el Ministerio de Salud y Protección Social y la Sociedad Colombiana de Urología recomiendan la detección temprana en hombres mayores de 50 años o menores de 50 años si se presentan factores de riesgo ^28^, ya que la evidencia científica ha mostrado mejores resultados del tamizaje en este grupo.

Con respecto a la mortalidad, en Colombia se han encontrado diferencias importantes en algunos departamentos como es el caso del Atlántico, al cual pertenece el Distrito de Barranquilla, donde las tasas de mortalidad por cáncer fueron superiores al promedio nacional ^4^; para el período 2007-2011 la tasa de mortalidad estandarizada por edad para cáncer de mama por 100,000 habitantes fue de 9.5 en el país, mientras que para el departamento del Atlántico este valor fue de 12.0, siendo superado solamente por Valle del Cauca con 12.3, en tanto que para próstata a nivel nacional el valor fue de 10.5 por 100,000 habitantes, y para el Departamento esta cifra fue de 14.6, ocupando el segundo lugar a nivel nacional ^4^.

En el presente estudio se encontró que las tasas para mama y próstata son las más altas: 15.7 y 17.4 por 100,000 habitantes en cada caso, tal como lo plantea el Análisis de Situación de Salud del Distrito de Barranquilla ^10^, el cual también indica que la tasa de mortalidad de cáncer de mama se ha ido incrementando progresivamente en 2.43 puntos del año 2004 al 2014, mientras que en ese mismo período próstata ha descendido 5.36 puntos.

Es de destacar que, el presente estudio es el primero que toma datos del Sistema de Información de Cáncer en Colombia para analizar mortalidad. Esta herramienta se encuentra disponible al público en general a partir del año 2017, y es resultado del esfuerzo del Instituto Nacional de Cancerología y los Registros de Cáncer de Colombia. El tomar esta fuente y no los DCO del DANE “directamente”, supone un reto interesante que permite vislumbrar el alcance y potencial del sistema de información como recurso importante para la toma de decisiones en el país. Se encontró que el cáncer de mama fue la primera causa de muerte entre las mujeres, y en los hombres la mortalidad por cáncer se atribuyó principalmente al de próstata.

## Conclusiones

La información obtenida por el RPCB es confiable, de acuerdo con los estándares requeridos por la IARC, y constituye una contribución importante al Sistema Nacional de Información del Cáncer. El comportamiento epidemiológico del cáncer en Barranquilla presenta variaciones con respecto a lo encontrado en el país y reportado en años anteriores, aunque el riesgo de desarrollar cáncer o morir debido se considera intermedio al ser comparado con las cifras reportadas por otros registros. 

Las estimaciones realizadas para el período quinquenal 2008-2012 servirán como línea de base para la construcción de las tendencias futuras. La información generada por el RPCB provee un valioso aporte a la construcción de información epidemiológica confiable para el país, concretamente en una ciudad representativa de la costa norte colombiana, por lo que se debe garantizar su sostenibilidad y mejorar cada día sus objetivos y estrategias a mediano y largo plazo.
